# The Resurrection of Mabrokan: Production of Multiple Cloned Offspring from Decade-Old Vitrified Tissue Collected from a Deceased Champion Show Camel

**DOI:** 10.3390/ani11092691

**Published:** 2021-09-14

**Authors:** Mohammad Shamim Hossein, Xianfeng Yu, Young-Bum Son, Yeon-Ik Jeong, Yeon-Woo Jeong, Eun-Ji Choi, Alex H. Tinson, Kuhad Kuldip Singh, Rajesh Singh, Al Shamsi Noura, Woo-Suk Hwang

**Affiliations:** 1 UAE Biotech Research Center, Al Wathba South, Abu Dhabi 30310, United Arab Emirates; shamim@uaebrc.ae (M.S.H.); xianfeng79@126.com (X.Y.); ybs@adbrf.org (Y.-B.S.); youniks@adbrf.org (Y.-I.J.); doctorj1@adbrf.org (Y.-W.J.); choi@uaebrc.ae (E.-J.C.); 2 Jilin Provincial Key Laboratory of Animal Model, College of Animal Science, Jilin University, Changchun 130062, China; 3 Hilli E.T. Cloning and Surgical Centre, Presidential Camels and Camel Racing Affairs, Al-Ain 17292, United Arab Emirates; heffoundation@hotmail.com (A.H.T.); kskuhad@hotmail.com (K.K.S.); rajeshsingh@gmail.com (R.S.); noura@precamels.ae (A.S.N.)

**Keywords:** cryopreserved tissue, somatic cell nuclear transfer, in vivo matured oocytes, in vitro matured oocytes, camels

## Abstract

**Simple Summary:**

Somatic cell nuclear transfer (SCNT) is a technique used to reproduce individuals from their somatic cell nucleus, which is commonly known as cloning. In SCNT, viable cell lines are usually established from living animals and preserved for future use. In the present study, tissues were collected and cryopreserved (rather than cells) from a suddenly deceased champion show camel. We established fibroblast cell lines from this decade-old vitrified tissue and used them as nuclear donors. Both the in vitro and in vivo matured oocytes were used to produce cloned embryos. Blastocysts were transferred to synchronized recipients to establish pregnancies. A total of 18 pregnancies (5 from in vitro matured oocytes and 13 from in vivo matured oocytes) were established, and 11 live offspring were born (2 from in vitro matured oocytes and 9 from in vivo matured oocytes). We concluded that fibroblast cell lines could be established from long cryopreserved tissues, and these cells could be used as nucleus donors to clone animals.

**Abstract:**

Somatic cell nuclear transfer (SCNT) provides a unique opportunity to reproduce animals with superior genetics. Viable cell lines are usually established from tissues collected by biopsy from living animals in the SCNT program. In the present study, tissues were collected and preserved from a suddenly deceased champion camel. We established cell lines from these decade-old tissues and used them as nuclear donors. After 42 h of in vitro maturation, 68.00 ± 2.40% of oocytes reached the metaphase II (M II) stage while 87.31 ± 2.57% in vivo collected oocytes were matured at collection (*p* < 0.05). We observed a higher blastocyst formation rate when in vivo matured oocytes (43.45 ± 2.07%) were used compared to in vitro matured oocytes (21.52 ± 1.74%). The live birth rate was 6.45% vs. 16.67% for in vitro and in vivo matured oocytes, respectively. Microsatellite analysis of 13 camel loci revealed that all the SCNT-derived offspring were identical to each other and with their somatic cell donor. The present study succeeded in the resurrection of 11 healthy offspring from the decade-old vitrified tissues of a single somatic cell donor individual using both in vitro and in vivo matured oocytes.

## 1. Introduction

Undoubtedly, the camelids are characterized by not only tremendously large-scale adaptation capacity but also high parameters related to their zootechnical performance in captivity. The aforementioned phenotypic traits predestine these pseudo-ruminant cloven-hoofed mammals to be multiplied by such modern assisted reproductive technologies (ARTs) as cloning by somatic cell nuclear transfer (SCNT). Nonetheless, achieving the satisfactory efficiency of intra- and inter-species somatic cell cloning both within the taxonomic family Camelidae and in other representatives of ruminant and non-ruminant artiodactyls requires comprehensively recognizing the epigenetic and molecular determinants of successfully generating SCNT-derived embryos, conceptuses, and progeny. Among those determinants, the most important ones appear to be the origin of the ex vivo-expanded nuclear donor cells (NDCs) [1,2,3,4,5], the approaches applied to ectopically synchronize the mitotic cycle of NDCs [6,7,8,9], and the attributes associated with molecular quality of extracorporeally proliferating NDCs. The latter can be negatively correlated with the frequencies of occurrence noticed for programmed cell death and DNA aneuploidy in NDCs and/or cloned embryos [10,11,12,13,14]. Furthermore, the pivotal factors determining the SCNT efficacy encompass epigenetic re-programmability of donor cell nuclear genomes [15,16,17,18,19] and their communication with mitochondrial DNA fractions [20,21,22,23,24] in SCNT-derived oocytes and corresponding embryos.

Since the production of the first SCNT-derived one-humped dromedary camel [25], several breeds or utility types of camels, including beauty, dairy, and racing camels, have been cloned for scientific and commercial purposes [26]. The two-humped Bactrian camel has also been produced by interspecies somatic cell nuclear transfer, using the dromedary camel as both the oocyte donor and surrogate [27]. The success of the SCNT procedure largely depends on the availability of viable donor cells. For the SCNT procedure, cell lines are usually established from tissues collected by biopsy and systemically preserved for future use. Similarly, all previous reports on camel cloning have used cryopreserved somatic cells as a nuclear donor [25,26,27]. 

The show camel, Mabrokan, was arguably the most well-known and historically valuable camel in the world. He won many beauty competitions over several years, and while living, remained a champion all the years he competed (Khaleej TimesDh15m Camel Does His Owner Proud by Winning Big Race-News|Khaleej Times). Mabrokan weighed over 1000 kg and had a massive head that towered 3 m above his handlers in his “show stance”. Mabrokan died unexpectedly in 2010 on a day where temperatures reached an excess of 50 °C. There was some foresight to potential use or recovery of the genetic material at the time of death. However, his carcass was in suboptimal condition for quite a long time before the practicing Veterinarian conduct skin and testicle biopsies. A decade later, the decision was made to attempt to recover viable cells from the long dead, but not forgotten Mabrokan and subsequently resurrect it by SCNT. 

Most SCNT studies use somatic cells line established from viable tissues as the integrity of the genome in the donor nuclei is essential for successful cloning [28]. However, the efforts that have been undertaken to generate SCNT-derived embryos and/or offspring with the use of somatic cells originating from tissues frozen in the absence of cryoprotectants have been successfully accomplished in the mice [29], domestic dogs [30], cattle [31], and cheetahs [32]. To date, no report on the cloning of camels from vitrified tissues is known. Here we reported the results of a study aimed at cloning a deceased camel from decade-old vitrified tissue.

## 2. Materials and Methods

### 2.1. Chemicals and Media

All chemicals and reagents were purchased from Sigma (St. Louis, MO, USA), unless otherwise noted. 

### 2.2. Oocyte Collection from Slaughterhouse Ovaries

Ovaries were collected from the local slaughterhouse and transported to the laboratory in lukewarm 0.9% saline solution. Antral follicles with a diameter of 2 to 6 mm were aspirated with an 18-gauge hypodermic needle attached to a 10 mL disposable syringe to collect cumulus oocytes complexes (COCs). COCs with dark and homogenous cytoplasm and having at least three layers of compact cumulus cells were selected for maturation. COCs were washed three times in Dulbecco’s phosphate-buffered Saline (DPBS; Welgene, Gyeongsan, KR) supplemented with 5 mg/mL bovine serum albumin (BSA; Thermo fisher scientific, Waltham, MA, USA) and 1% antibiotic-antimycotic (Thermo fisher scientific, Waltham, MA, USA). The selected COCs were cultured at 38 °C in 5% CO_2_ in a humidified atmosphere for 42 h in a commercial IVM media (IVF Bioscience, Falmouth, UK).

### 2.3. Care and Management of Camel

Experiments were conducted during the local breeding season of the camel, November 2019 to February 2020. Pregnant camels were maintained in the research facility until parturition. Camels with normal breeding history and without any abnormalities in the reproductive tract were selected and used as oocyte donors and surrogates. Animals were daily fed appropriate nutrients and water was given ad libitum. A total of 102 females (17 egg donors and 85 recipients) that were aged 4–7 years and weighed 400–450 kg were used in this study.

For ovarian stimulation, a single intramuscular injection of 5000 IU PMSG (Ceva, Libourne, France) and 500 µg of Closprostenol (Jurox, Rutherford, Australia) was given to the donor camels and this day is considered as Day 0. The recipients also received a single intramuscular injection of 1500 IU PMSG and 100 µg of Closprostenol on the same day. On Day 9, both the donors and recipients were evaluated for super-ovulatory response by ultrasonography. Camels having at least five follicles with a diameter of 10 mm to 20 mm were finally selected as a donor. Intramuscular injection of 100 µg Gonadorelin Acetate (Vetoquinol, Paris, France) was given to donors for the final maturation of oocytes. After 25 to 28 h of injection OPU was performed. The recipient has also received 100 µg of Gonadorelin Acetate for ovulation and corpus leutium (CL) formation on Day 9.

### 2.4. Collection and Cryopreservation of Mabrokan Tissue

Postmortem skin tissues were collected aseptically and vitrified following the previously described report [33]. Briefly, tissues were washed three times with DPBS (Life Technologies, Carlsbad, CA, USA) containing 1% (*w*/*v*) penicillin-streptomycin solution (Invitrogen). After that, tissues were cut into small pieces (1 cm^3^) using surgical blades at room temperature. The pieces were transferred to Dulbecco’s modified Eagle’s medium (DMEM; Thermo Fisher Scientific, Waltham, MA, USA) supplemented with 20% ethylene glycol (EG) and 10% fetal bovine serum (FBS; Invitrogen, Waltham, MA, USA) for 1 min and subsequently transferred to DMEM supplemented with 40% EG and 10% FBS into cryo-vials (Nunc, Roskilde, Denmark). The tissues were then immediately transferred to liquid nitrogen (−196 °C).

### 2.5. Establishment of Skin Fibroblast Cell Line

The vitrified tissue was thawed as previously reported with minor modifications [33]. Briefly, to remove the cryoprotectant, tissues were kept at room temperature for 5 min then transferred to DMEM supplemented with 0.3 M sucrose and 10% FBS at 38 °C for 5 min followed by DMEM supplemented with 0.15 M sucrose and 10% FBS for 5 min. Tissues were then washed 3 times with DMEM supplemented with 10% FBS. After that, samples were minced into small pieces with a scissor and digested in DMEM supplemented with 0.1% collagenase type II (Thermo Fisher Scientific, Waltham, MA, USA) at 38 °C in a humidified atmosphere with 5% CO_2_ for 2 h. The dispersed cells were washed with DMEM by centrifugation at 300× *g* for 5 min and filtered through a 40 µm nylon strainer (Falcon, Franklin, NJ, USA). The cell pellets were cultured in DMEM supplemented with 10% FBS (Thermo Fisher Scientific, Waltham, MA, USA), 1% nonessential amino acid (Thermo Fisher Scientific, Waltham, MA, USA), 1% antibiotic-antimycotic (Thermo Fisher Scientific, Waltham, MA, USA), and 0.1% β-mercaptoethanol (Thermo Fisher Scientific, Waltham, MA, USA) at 38 °C in a humidified atmosphere with 5% CO_2_. The culture media was changed every two days until confluence reached 80% and passaged using 0.25% trypsin EDTA solution.

### 2.6. Somatic Cell Nuclear Transfer (SCNT)

SCNT was performed following previously described techniques with minor modifications [25]. In brief, cumulus cell layers were removed from the oocytes by gentle pipetting with 0.1% hyaluronidase. After being denuded, M II oocytes were stained with 5 μg/mL bisbenzimide for 3 min to detect the genetic materials. The nucleus and polar bodies were aspirated from the oocytes and a single fibroblast cell was microinjected into the perivitelline space of the oocytes. Non-starved donor cells at their early passage (3 to 5 passage; 70% to 90% confluent) were used as donor nucleus. After that, these reconstructed oocytes were fused in fusion media composed of 0.26 M mannitol, 0.1 mM MgSO_4_, 0.5 mM HEPES, and 0.05% (*w*/*v*) BSA with two DC pulses of 1.8 kV/cm for 15 μsec using BTX Electro Cell Manipulator (BTX Inc., San Diego, CA, USA). After that, the fused oocytes were activated chemically using 5 μM ionomycin for 3 min and 2.0 mM 6-dimethylaminopurine (6-DMAP) in BO-IVC (IVF Bioscience, Falmouth, UK) in a humidified incubator of 5% CO2 at 39 °C for 4 h.

### 2.7. Embryo Culture and Transfer to the Recipient

After activation, reconstructed oocytes were cultured in a commercial embryo culture media, BO-IVC. A group of 6 to 8 oocytes were cultured in a 30 μL oil-covered droplet at 38 °C in a humidified atmosphere with 5% CO_2_ and 5% O_2_. Embryos were evaluated at 2 and 7 days for developmental competency to cleavage and blastocyst stage. On day 7, blastocysts were transferred transvaginally to the left horn of synchronized recipients.

### 2.8. Pregnancy Diagnosis

Serum progesterone level was measured using Chemiluminescence Immunoassay (Roche, Basel, Switzerland) after 16 days of blastocyst transfer. An initial rise of serum progesterone level to >1 ng/mL was considered as pregnant. On day 30, real-time ultrasonography was performed at a standing position to confirm the pregnancies. Second ultrasonography was performed 90 days after ET.

### 2.9. Microsatellite Analysis

Cloned calf parentage was confirmed using the standard procedure of short tandem repeats (STR) profiling. Microsatellite analysis was carried out using 13 specific loci for *Camelus dromedarius*, as shown in Table 1. The genomic DNA was isolated from individual donor cells, venous blood of cloned calves, and recipients using the DNA isolation kit from Qiagen (Qiagen DNeasy Blood and Tissue kit; Qiagen, Hilden, Germany).

### 2.10. Statistical Analysis

Statistical analysis was performed using SPSS (version 15; SPSS Inc., Chicago, IL, USA). The student’s *t*-test was performed to analyze the differences in the development of embryos between the groups. Pearson Chi-square test and Fisher’s exact test were conducted to compare the pregnancy rates. Data were represented as mean ± standard error (SE) and *p* values less than 0.05 were considered statistically significant.

## 3. Results

### 3.1. Effect of the Source of Oocytes on Oocyte Maturation 

**Figure 1:**
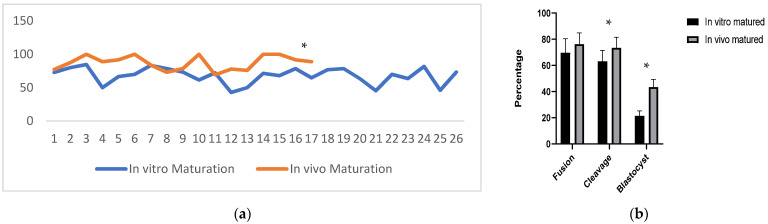
(**a**). In vivo and in vitro maturation rate of camel oocytes. (**b**) Developmental rate of SCNT-derived camel embryos using in vitro and in vivo matured oocytes, * in the same group indicates a significant difference (*p* < 0.05).

A total of 348 COCs were collected from 26 ovaries obtained from the local slaughterhouse, and 68.00 ± 2.40% of oocytes reached to metaphase II (M II) stage after 42 h of IVM (Figure 1a). A total of 292 oocytes were collected from 17 camels that received superstimulation treatment by the ovum pick up (OPU) method. The mean oocyte number of oocytes per camel was 17.18 ± 1.98% while 87.31 ± 2.57% was at the MII stage during collection. We observed significantly (*p* < 0.05) higher maturation potential of the collected oocytes derived from OPU compared with oocytes from the slaughterhouse. 

### 3.2. Developmental Competence of SCNT-Derived Embryos

**Figure 2:**
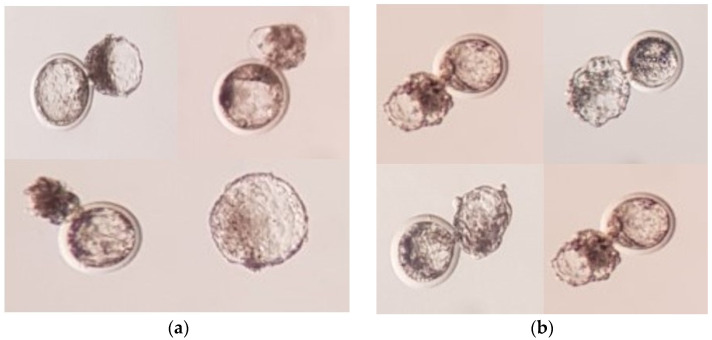
Representative photographs of blastocysts produced by SCNT using (**a**) in vitro matured oocytes (**b**) in vivo matured oocytes.

The developmental competence of reconstructed embryos with in vitro or in vivo matured oocytes is presented in Figure 1b. The percentage of fused oocytes was 61.52% and 69.31% in in vitro and in vivo matured oocytes group, respectively. These differences were not significant (*p* > 0.05). The percentage of cleaved oocytes was 62.12% and 78.31%, and the blastocyst formation rate was 21.52% and 43.45% in in vitro and in vivo matured oocytes group, respectively (*p* < 0.05). The representative photograph of blastocysts produced by in vitro and in vivo matured oocytes are shown in Figure 2.

### 3.3. Efficiency of Pregnancy Rate and Parental Analysis of Cloned Camel

**Figure 3:**
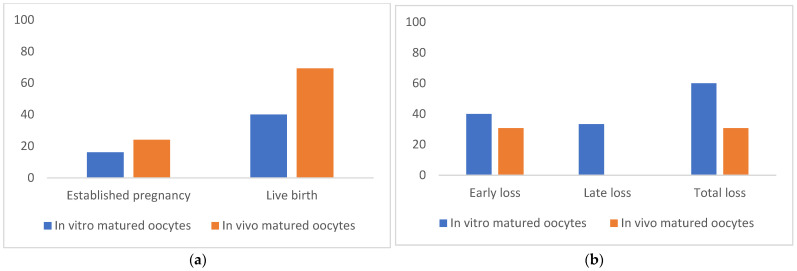
**(a).** Pregnancy and live birth rate following SCNT-derived ET **(b)** Pregnancy loss following SCNT-derived ET. The differences were not significant (*p* > 0.05).

A total of 61 blastocysts derived from in vivo-matured oocytes were transferred to 54 surrogates, and 35 blastocysts derived from in vitro-matured oocytes were transferred to 31 recipients. Among the 54 surrogates in the in vivo matured oocytes group, 13 (24.07%) pregnancies were detected, and 9 (16.67%) live births were obtained. Among the 35 recipients in in vitro maturation group, 5 (16.12%) pregnancies were detected, and 2 (6.45%) live births were obtained (Figure 3a). There was no significant difference in the efficiencies of pregnancy and live birth in the in vivo-matured oocyte group compared with the in vitro matured group.

Among the 5 pregnancies in the in vitro group, 2 (40%) pregnancies were lost before 90 days of pregnancy and 1 (20%) late abortion was observed at 24 weeks of pregnancy (Figure 3b). Among the 13 pregnancies in the in vivo group, 4 (30.76%) pregnancies were lost before 90 days of pregnancy, and no late abortion was observed in this group. 

**Figure 4:**
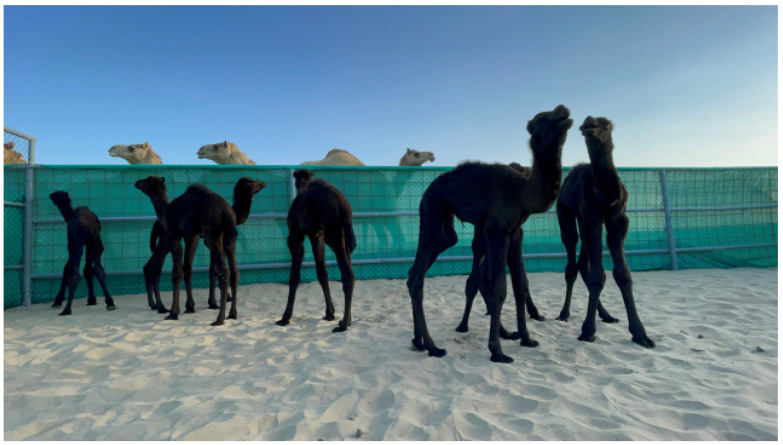
Representative photograph of cloned offspring.

Microsatellite analysis of 13 camel loci revealed that all the SCNT-derived offspring were identical to each other and with their somatic cell donor (Table 2). The representative photograph of cloned offspring is shown in Figure 4.

## 4. Discussion

In the present study, we have demonstrated that healthy cloned offspring could be obtained by nuclear transfer using donor nuclei obtained from decade-old vitrified tissues of a suddenly deceased champion show camel named Mabrokan. Generally, tissue biopsy is taken ante-mortem and cell lines are established and cryopreserved at their early passage for future use as a nuclear donor in the SCNT program [25,26,27]. Here, we established a primary fibroblast cell line from deceased and vitrified dermal tissues and produced 11 viable offspring from those somatic cells. To our best knowledge, this is the first large-scale report of a cloned camel from cryopreserved tissue collected from a single deceased animal. 

The developmental capacity of reconstructed embryos is influenced by the intrinsic quality of oocytes [34]. Proper nuclear and cytoplasmic maturation is the key element of a good quality oocyte. In vivo matured oocytes are generally considered superior in quality compared with in vitro matured oocytes, as they contain a higher concentration of glutathione, which is an important indicator of cytoplasmic maturation [35,36]. We have observed a greater blastocyst formation rate (43.45%) when in vivo matured oocytes were used compared to in vitro matured oocytes (21.52%). In a previous study involving camels, Wani et al. [9] reported greater cleavage and blastocyst production rate following SCNT using in vivo matured oocytes compared with in vitro matured oocytes. Akagi et al. [37] reported an improvement of developmental competence of bovine SCNT-derived embryos by using in vivo-matured oocytes. These low developmental competencies of in vitro matured oocytes may be due to the inferior quality of cytoplasmic maturation. However, the cellular mechanism involved in cytoplasmic maturation in the oocyte is unclear. Maturation medium or intrinsic quality of oocytes or both may be responsible for improper cytoplasmic maturation and decreased potential of embryonic development [38].

In vitro developmental parameters of reconstructed embryos such as blastocyst production rate significantly varies according to the source of oocytes, no differences were observed in the efficiency of pregnancy and live birth rates. The mean live birth rate ranges from 6.45% to 16.67% in different groups. This cloning efficiency is comparable to that using cell lines established using live normal tissues [25,26,27]. We have reported to our earlier study that fibroblast cell lines established from vitrified tissues showed similar growth patterns, apoptosis rate, and mitochondrial metabolism with cells from fresh tissues [39]. Furthermore, the blastocyst production rate after SCNT was also similar in both groups.

A higher rate of pregnancy loss compared to other domestic animals was observed in the present study. Pregnancy loss varied from 30.76% to 60.0% in in vivo and in vitro matured oocyte groups. Wani et al. [25] reported a higher rate of pregnancy losses (33% to 100%) within the first 3 months of pregnancy following transfer of blastocyst produced by SCNT using different cell lines. Even in natural breeding higher incidence of early embryonic loss (30% to 40%) was reported in camels [40]. No pregnancy loss was observed after 90 days of pregnancy in in vivo matured oocyte group, whereas 20% (1 out of 5 pregnancies) abortion was observed at 168 days of gestation. Vettical et al. [41] reported a similar pattern of pregnancy loss in camel following the transfer of SCNT-derived embryos; they observed 18% pregnancy loss within 60 days of pregnancy, and 7% (1 out of 15 pregnancies) of pregnancy loss after 90 days of gestation. Determine the exact causes of higher embryonic loss in camel is difficult, as numerous factors may complicate the diagnosis. However, the intrinsic quality of reconstructed embryos, abnormal epigenetic reprogramming of somatic cell nucleus, chromosomal abnormalities, and luteal deficiency in surrogates may be the important causes of pregnancy loss in camel [42].

## 5. Conclusions

The present study succeeded in the resurrection of 11 healthy offspring from decade-old vitrified tissues of a single somatic cell donor individual. Although the in vivo and in vitro matured oocytes differ in the ability to generate blastocysts, the source of oocytes does not appear to differ in the production of healthy offspring, hence in vivo and in vitro oocytes could be used in camel cloning.

## Figures and Tables

**Table 1:** Characteristics of 13 microsatellite loci for *Camelus dromedarius*.

Markers	Allele Range	doi
VOLP10	240–269	https://doi.org/10.1046/j.1365-2052.1999.00526-19.x
VOLP67	145–208	https://doi.org/10.1046/j.1365-2052.1999.00526-19.x
LCA63	198–232	https://doi.org/10.1046/j.1365-2052.1999.00382-8.x
LCA66	224–242	https://doi.org/10.1046/j.1365-2052.1999.00382-8.x
LCA90	234–246	https://doi.org/10.1046/j.1365-2052.1999.00526-21.x
CVRL01	188–253	https://doi.org/10.1046/j.1365-2052.2002.00896_6.x
CVRL05	155–185	https://doi.org/10.1046/j.1365-2052.2002.00896_6.x
CVRL07	270–230	https://doi.org/10.1046/j.1365-2052.2002.00896_6.x
LGU49	224–260	https://doi.org/10.1046/j.1365-294x.2000.01077-3.x
LGU75	184–230	https://doi.org/10.1046/j.1365-294x.2000.01077-3.x
YWLL44	86–120	https://doi.org/10.1111/j.1365-2052.1996.tb00502.x
P149	256–284	https://doi.org/10.1016/j.smallrumres.2009.07.012
PCTD17	172–204	https://doi.org/10.1016/j.smallrumres.2009.07.012

**Table 2:** Microsatellite analysis of donor cells, cloned offspring, and surrogates.

Markers	Donor Cells	Cloned Offspring	Surrogates
VOLP10	259/265	259/265	249/259, 251/259, 249/259, 249/249, 249/259, 249/251, 251/259, 251/251, 249/259, 249/251, 249/259
VOLP67	147/147	147/147	178/178, 155/186, 176/190, 176/186, 153/192, 153/153, 178/188, 153/155, 155/188, 153/178, 153/155
LCA63	212/220	212/220	216/220, 214/216, 216/218, 216/220, 214/220, 212/214, 218/220, 216/220, 214/220, 214/220, 214/220
LCA66	240/240	240/240	238/240, 238/240, 238/240, 238/238, 234/234, 234/240, 234/238, 238/240, 238/238, 234/238, 234/238
LCA90	238/240	238/240	240/242, 240/240, 238/238, 240/240, 240/240, 240/240, 240/240, 238/240, 240/242, 238/240, 240/240
CVRL01	228/234	228/234	204/234, 226/234, 212/214, 202/214, 212/214, 218/224, 226/246, 234/234, 234/234, 228/228, 214/214
CVRL05	163/171	163/171	159/169, 171/179, 159/171, 159/159159/169, 159/159, 159/171, 171/171, 159/159, 159/171, 159/169
CVRL07	295/295	295/295	285/285, 281/285, 285/285, 273/277, 273/277, 273/281, 295/295, 281/285, 281/281, 273/273, 277/277
LGU49	223/235	223/235	223/239, 239/242, 239/242, 221/231, 225/225, 223/229, 223/225, 225/225, 223/225, 225/229, 225/229
LGU75	188/226	188/226	194/204, 204/228, 188/204, 192/204, 192/204, 204/224, 188/230, 208/208, 188/230, 188/204, 192/224
YWLL44	148/167	148/167	135/179, 163/169, 135/163, 148/176, 135/135, 135/163, 135/163, 135/135, 135/167, 135/167, 148/163
P149	268/284	268/284	260/284, 260/284, 268/268, 260/260, 260/268, 260/284, 260/268, 260/284, 260/284, 260/260, 260/268
PCTD17	184/184	184/184	184/192, 188/192, 188/192, 192/192, 184/192, 188/192, 184/188, 188/188, 184/188, 184/188, 192/192

Microsatellite analysis was performed on genomic DNA from cloned offspring, surrogate, and donor cells. The values of all markers were confirmed identically in all cloned offspring. Values represent the base pairs of the amplified microsatellite DNA markers in each sample.

## Data Availability

Data access can be requested on demand from the corresponding author.

## Abbreviations

Abbreviation	Meaning
6-DMAP	6-dimethylaminopurine
BSA	Bovine serum albumin
CL	Corpus leutium
COC	Cumulus oocytes complex
DMEM	Dulbecco’s modified Eagle’s medium
DPBS	Dulbecco’s phosphate-buffered saline
EG	Ethylene glycol
FBS	Fetal bovine serum
NDC	Nuclear donor cell
SCNT	Somatic cell nuclear transfer
